# Operative Fixation of a Lateral Acromion Fracture in a Native Shoulder Using Nice Knot Suture Cerclage

**DOI:** 10.1002/atn2.70070

**Published:** 2026-07-19

**Authors:** Garrett V. Christensen, Cory J. Call, Michael C. Marinier, Reilly T. Corken, Maria F. Bozoghlian, Carter Lane, James V. Nepola, Brendan M. Patterson, Joseph W. Galvin

**Affiliations:** ^1^ Department of Orthopedics and Rehabilitation University of Iowa Health Care North Liberty Iowa U.S.A.

## Abstract

Acromion fractures are challenging injuries to manage. Nonoperative treatment for nondisplaced fractures can yield satisfactory outcomes for most patients. Displaced acromion fractures can be difficult to manage given the vector of displacement and limited bone for fixation. There is no consensus on surgical approach to these fractures as they are rare. Acromion fractures in native shoulders are rarer as most acromion fractures are secondary to altered mechanics and decreased bone mineral density following reverse shoulder arthroplasty. The acromion is a thin and flat bone making plate and screw fixation challenging. This technical note describes a technique for open reduction and internal fixation of a lateral acromion fracture in a native shoulder utilizing Nice knot suture cerclage.

## Graphical abstract

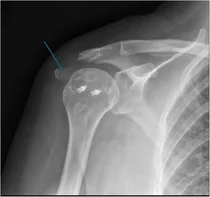

**VIDEO 1:** Video showing the surgical technique on a simulated lateral acromial fracture in a cadaveric specimen. Video content can be viewed at https://doi.org/10.1002/atn2.70070.

Scapula fractures are an uncommon injury, comprising approximately 1% of all fractures.^1^ With muscles encasing the scapula in addition to its mobility with respect to the thorax, surrounding musculoskeletal structures such as the clavicle or proximal humerus tend to succumb to injury first.^2^ Of reported scapular fractures, approximately 8% involved fractures of the acromion process. Acromion fractures are generally caused by direct, high‐energy trauma to the shoulder girdle and are typically associated with concomitant injuries due to the mechanism of impact.^3^, ^4^ Additional injury mechanisms of acromion fractures consist of avulsion fractures induced by muscles and insufficiency fractures after reverse shoulder arthroplasty (RSA) (Figures 1 and 2).^5^, ^6^, ^7^, ^8^

**FIGURE 1:**
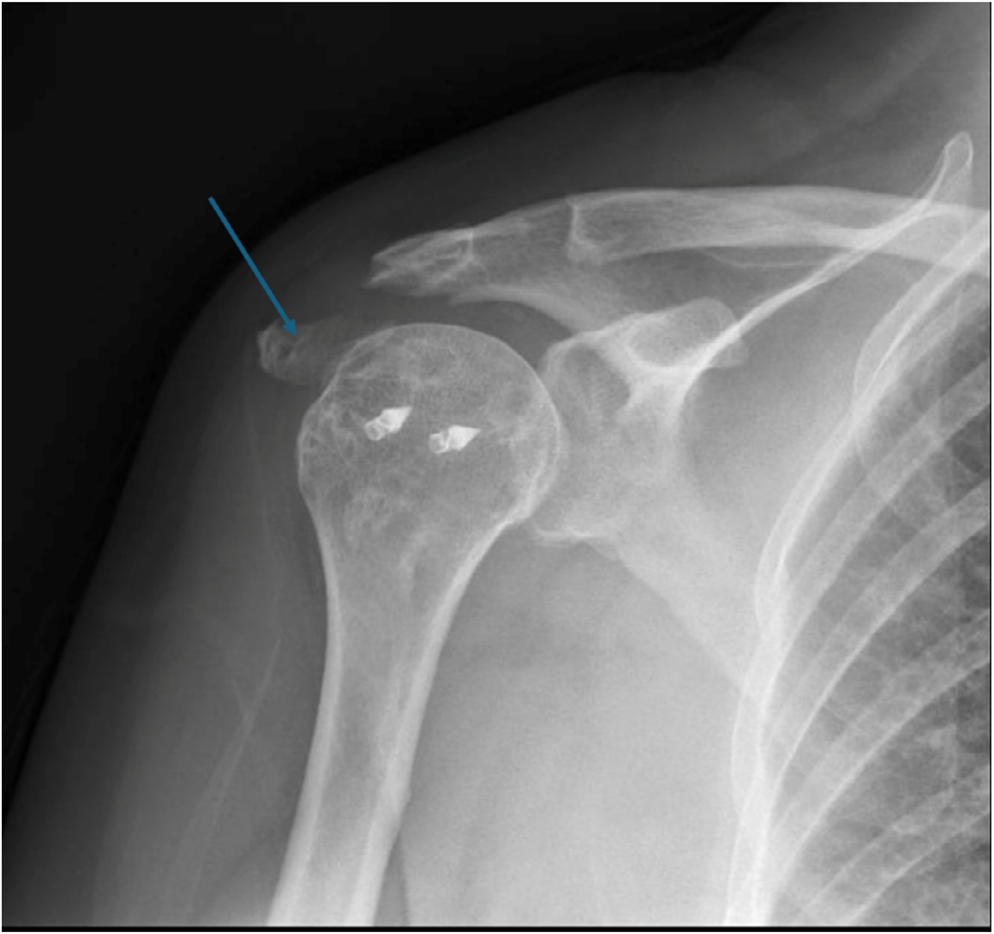
Preoperative radiograph of the right shoulder showing a displaced lateral acromion fracture (blue arrow). There are anchors visible from a prior arthroscopic rotator cuff repair.

**FIGURE 2:**
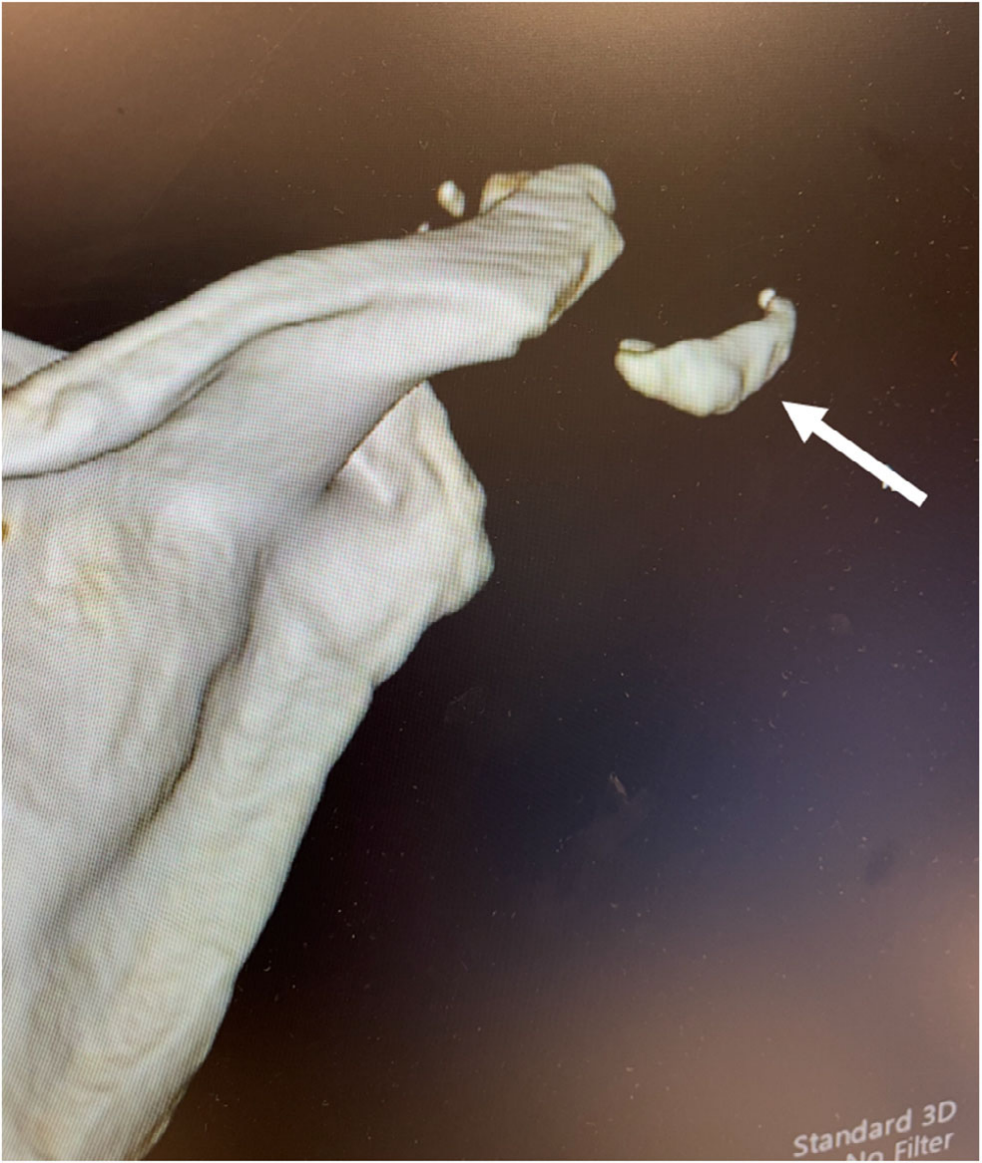
Preoperative 3D CT scan showing a right shoulder displaced lateral acromion fracture (white arrow) with a fracture line running in an anterior‐to‐posterior direction. This fracture fragment has a large portion of the anterior and middle deltoid attached to it.

In 1994, Kuhn et al.^9^ described a classification system for fractures of the acromion (Figure 3). Type I fractures are defined by a minimally displaced acromion and are subdivided into two types to differentiate the mechanism of injury. Type IA fractures are atraumatic or avulsion fractures, are the result of excessive strain placed on the acromion by the deltoid. Type IB fractures occur after direct trauma to the shoulder and are described by displacement of the acromion fracture less than 2 mm. Fractures that are displaced superiorly, anteriorly, or laterally, and do not decrease the subacromial space are classified as Type II. Type III fractures lead to inferior displacement of the acromion process and obliteration of the subacromial space.^9^

**FIGURE 3:**
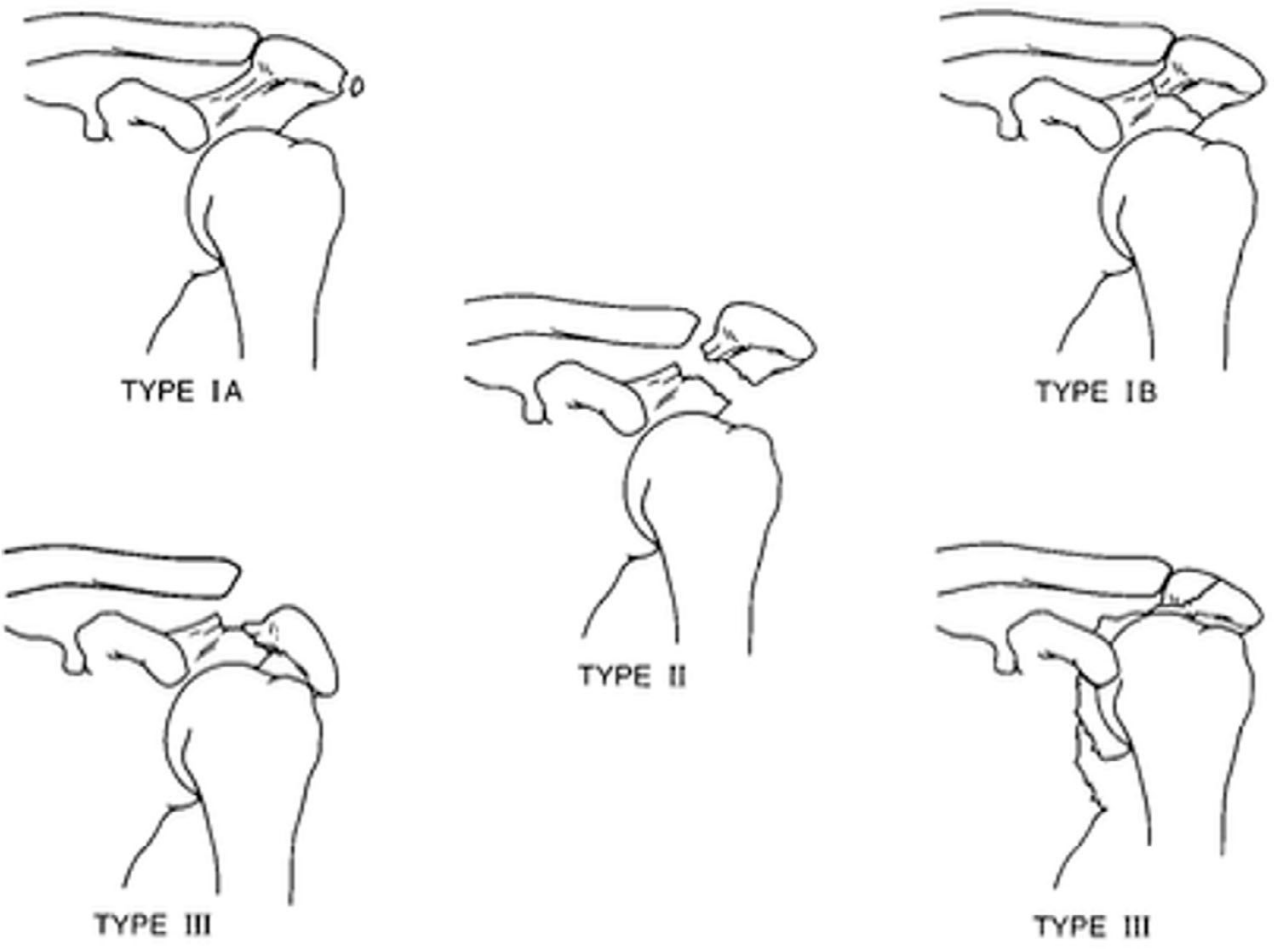
Kuhn classification of acromion fractures.

Serving as the attachment for the coracoacromial ligament, acromioclavicular ligament, deltoid, and trapezius, the acromion process is pivotal in providing shoulder stability and mobility.^10^ Without an intact acromion, the shoulder can be dysfunctional as flexion and abduction of the shoulder is limited.

There is no standardized treatment protocol for these fractures and there is minimal evidence describing the indications for conservative or operative treatment.^11^ Acromion fractures in native shoulders are rarer as most acromion fractures are secondary to altered mechanics and decreased bone mineral density following RSA.^10^ Some studies suggest surgical intervention through open reduction and internal fixation (ORIF) have generated adequate outcomes for displaced acromion fractures.^11^ The objective of ORIF is to anatomically reduce the lateral fragment of the acromion, restore the subacromial space, create robust fixation to neutralize the forces of the deltoid, and to promote bone healing through compression of the fracture. There are several techniques described for ORIF of acromion fractures, most commonly including K‐wires, tension band techniques, or plates and screws.^10^ There is no optimal fixation method due the thin nature of the acromion and the small bone fragments produced from acromial fractures. This technical note describes a technique for the ORIF and Nice knot suture cerclage for lateral acromion fractures.

## SURGICAL TECHNIQUE

### Step 1: Setup and Patient Positioning

**FIGURE 4:**
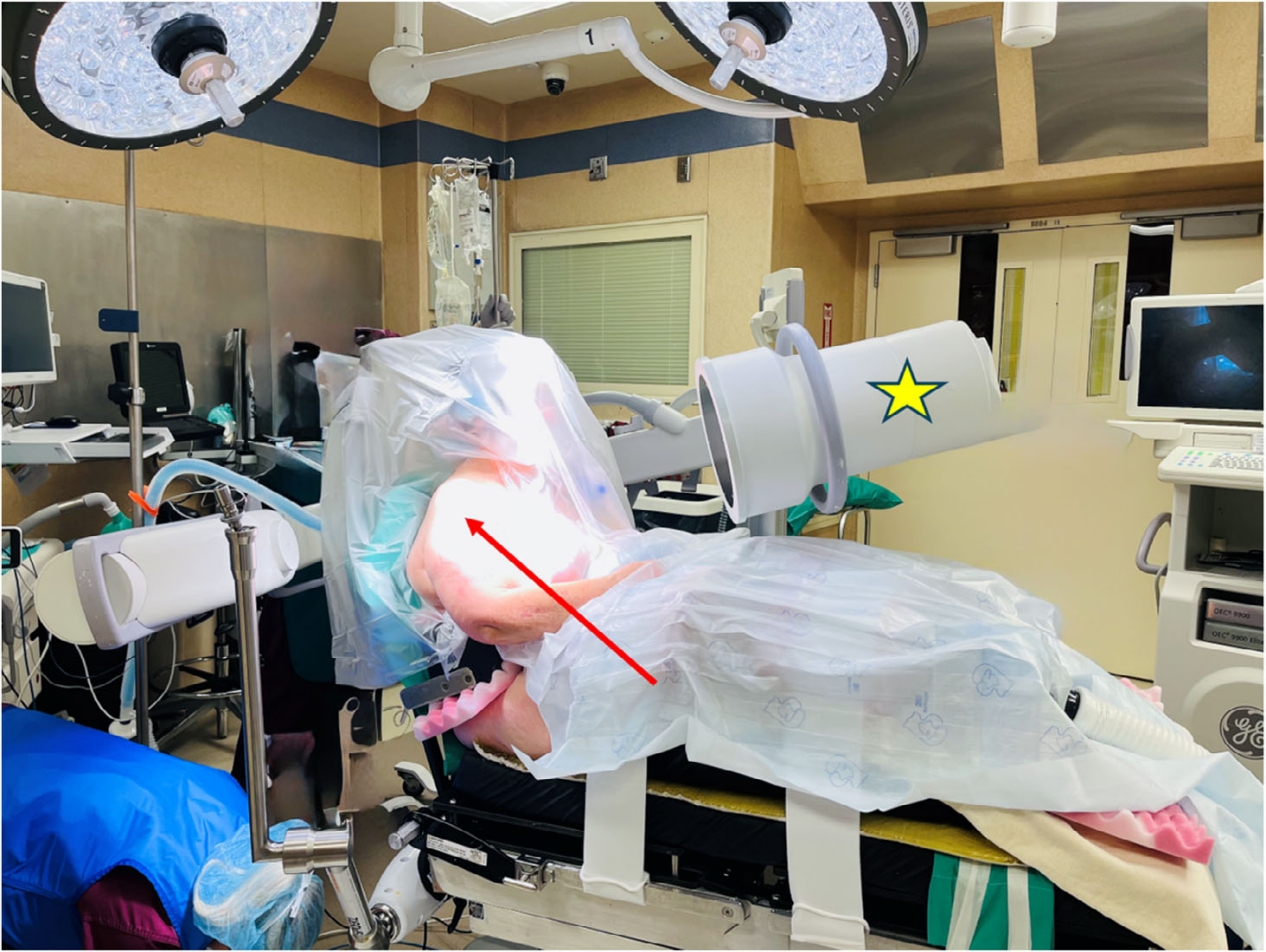
Intraoperative positioning of the patient's right shoulder (red arrow) in a modified beach chair position with large C‐arm (yellow star) from the contralateral side.

After general anesthesia and regional nerve block, the patient is positioned in the beach chair position. The senior author's preference is beach chair position; however, lateral decubitus position can be considered based on surgeon preference. All bony prominences are well padded. Large C‐arm fluoroscopy is brought in from the contralateral side of the bed, and preoperative imaging is performed to ensure adequate radiographic visualization of the fracture and the acromion (Figure 4). The patient undergoes sterile preparation and draping, and a timeout is performed confirming the operative site and delivery of perioperative antibiotics.

### Step 2: Acromion Exposure

**FIGURE 5:**
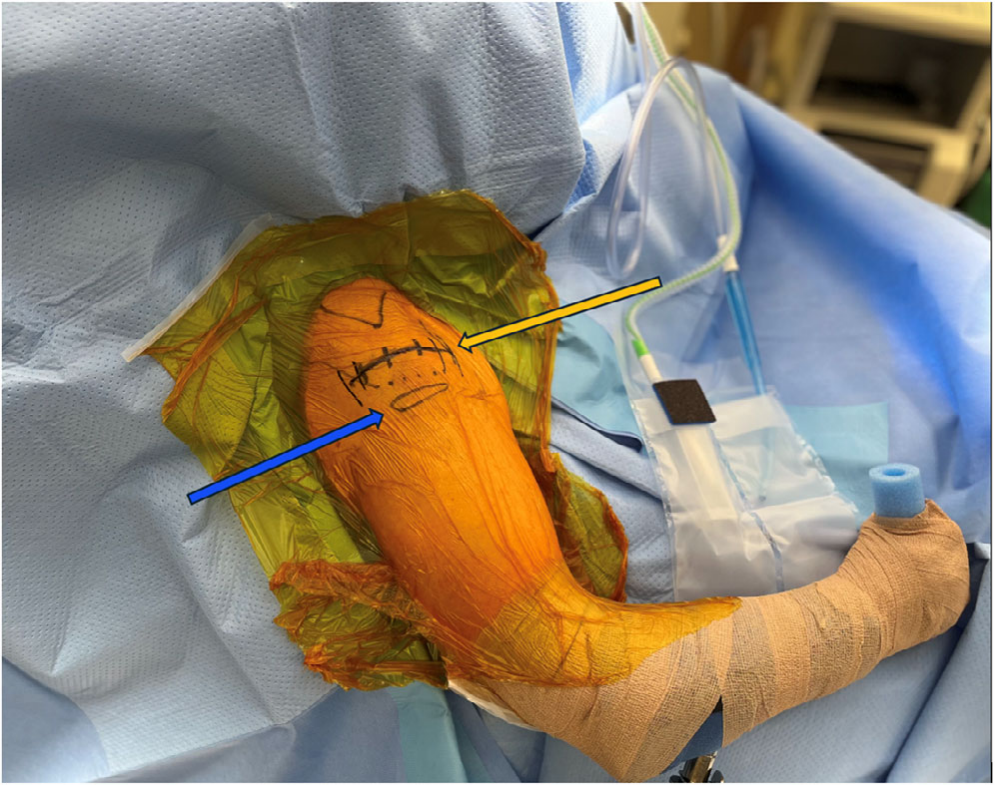
This is a photo of a right shoulder in the beach chair position. The incision (yellow arrow) is placed just medial to the lateral edge of the intact acromion. The displaced fracture fragment is palpable below the skin and is marked with a marking pen (blue arrow).

A saber‐type incision is made over the fracture site, or just medial to the fracture site to ensure adequate exposure of both the displaced fracture fragment as well as the medial side of the acromion near the Neviaser's portal area (Figure 5, Video 1). When the displaced fracture fragment is identified, a traction suture can be placed around it to aid in exposure and lysis of adhesions that may be restricting reduction of the fragment. Care should be taken to avoid injury to the deltoid muscle or tendon attached to the fracture fragment. The arm can also be abducted to aid in reduction of the fracture.

### Step 3: Fracture preparation

When the displaced fragment is freed and a traction stitch is placed, the medial, intact acromion should be exposed. A knife or curette should be used to biologically prepare the fracture site to aid in union after reduction.

### Step 4: Passage of Suture Tapes

**FIGURE 6:**
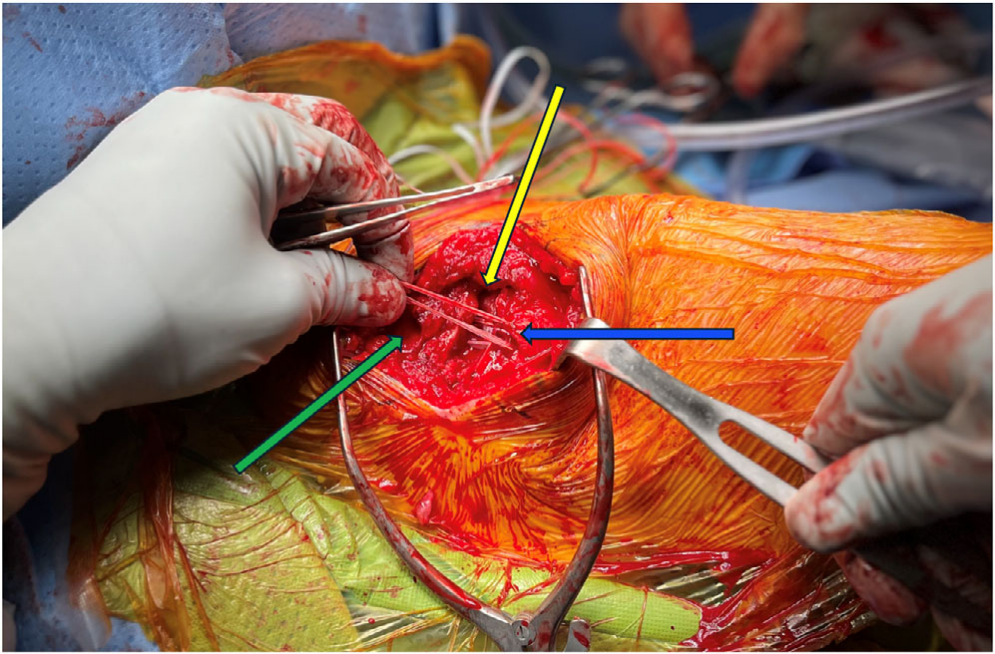
Intraoperative photo of the right shoulder from posterior in beach chair position. The fracture (yellow arrow) is visible and runs in an anterior to posterior direction. The displaced fracture fragment (blue arrow) is reduced to the intact acromion (green arrow) with multiple Nice loop suture cerclages.

Multiple 2.2‐mm looped sutures (Nice Loops, Stryker, Kalamazoo, MI) should be passed for later reduction and fixation (Figure 6). Our preferred technique is to pass the Nice loops from superficial to deep just lateral to the displaced fracture fragment through deltoid tendon, under the fracture fragment into the subacromial space, and from deep to superficial in the Neviaser's portal area. This step is repeated multiple times moving anterior and posterior until it is felt that there are adequate Nice loops to hold the reduction. It is important to pass enough cerclage sutures to distribute force evenly across the relatively thin bony fracture fragment to avoid pull out and breakage (Table 1).

**TABLE 1:** Pearls and Pitfalls

Pearls	Pitfalls
Shoulder abduction during the case aids in fracture reduction. This can be done with an assistant, an articulating arm holder, or a padded Mayo stand	Ensure adequate number of suture tapes are passed to distribute force evenly across the relatively thin acromial fracture fragment
Several of the Nice Loops can be tied simultaneously to distribute force more evenly at time of reduction and fixation	Postoperative shoulder abduction splint placement is critical to offload the fixation construct

### Step 5: Reduction and Fixation

**FIGURE 7:**
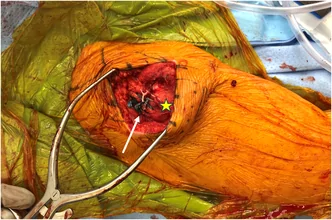
Intraoperative photo from superior of the right shoulder with patient in beach chair position. The displaced acromion fracture (yellow star) is reduced and secured with multiple Nice loops and Nice knots (white arrow).

**FIGURE 8:**
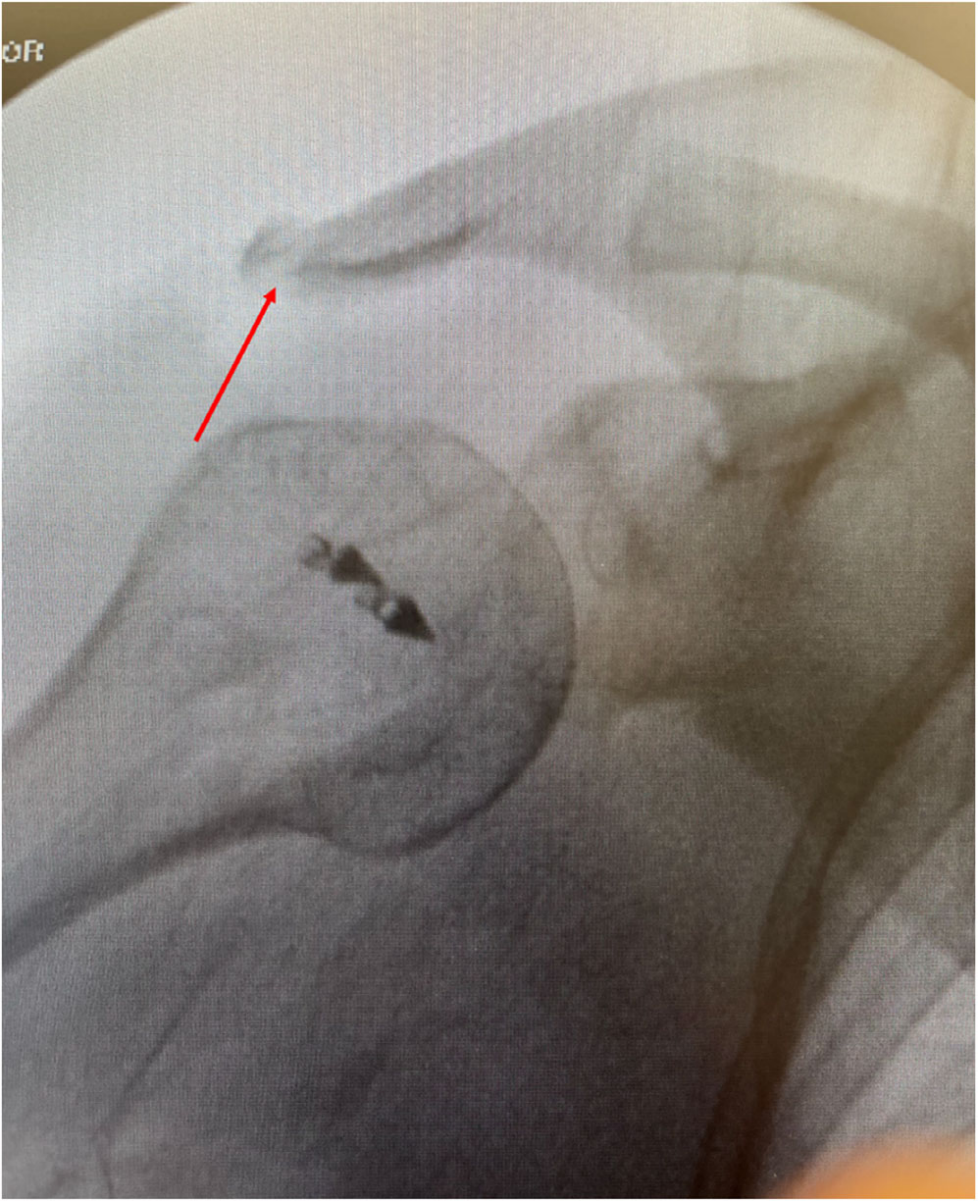
Intraoperative fluoroscopic image showing reduction of the displaced acromion fracture (red arrow).

Each looped suture tape is then tied with a Nice knot, as described previously.^12^ The Nice knot is a sliding, self‐locking knot that is ideal for holding a preliminary reduction as the knot provides enough tension to hold reduction prior to final tying. Our preferred technique is to perform the Nice knot in each of the looped suture tapes first, and only when all the tapes have been tightened should each knot be locked with 5 surgeon's knots (Figure 7). Final fluoroscopic images show maintained reduction (Figure 8).

### Step 6: Closure

The wound is irrigated and closed in layers. A sterile dressing is applied, and the patient is placed into an abduction brace in approximately 70‐80 degrees of shoulder abduction.

### Postoperative Care

**FIGURE 9:**
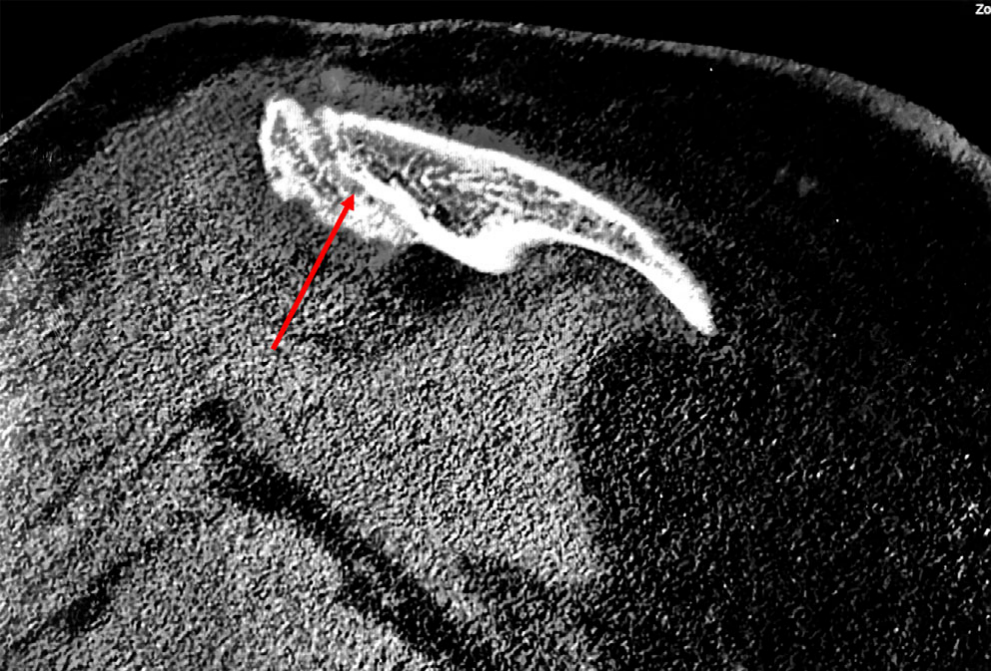
Six‐month postoperative coronal CT image showing bony union at the right acromial fracture site (red arrow).

Postoperatively, patients are non weightbearing with no shoulder range of motion in the abduction brace for approximately 6 weeks. They are then transitioned to a shoulder abduction immobilizer for an additional 6 weeks. At 3 months, the immobilizer is discontinued, and a CT scan is obtained to confirm healing at the fracture site (Figure 9). We obtain a CT scan as fracture union is difficult to discern on standard shoulder radiographs.

## DISCUSSION

Acromion fractures are rare, especially in native shoulders as many of these fractures are thought to be stress‐related fractures following RSA. In fractures of the native shoulder, acromion fractures are classified by Kuhn et al.^9^ both by injury mechanism and degree of displacement. Many of these fractures can be treated nonoperatively if the fracture fragment is minimally displaced and the subacromial space is maintained. For more displaced fractures, especially those with displacement into the subacromial space, open reduction internal fixation should be considered to restore the continuity of the deltoid. Given the rarity of these fractures, there is no consensus on optimal method for fixation. Treatment with Kirschner wires, plates and screws, fragment excision, and suture fixation have all been described and each have advantages and disadvantages. Plate and screw fixation for lateral acromion fractures is challenging given the flat morphology of the bone and the significant deforming force of the deltoid.

The current technical note provides an option for ORIF of a lateral displaced acromion fracture with Nice loop suture cerclage fixated with Nice knots. The main advantage of this technique is that it avoids the need for plate or screw fixation which is not ideal for small lateral acromial fracture fragments (Table 2). Additionally, it obviates the need for a second surgery for implant removal if the plate/screw construct becomes symptomatic. Also, in the event that the fixation fails, no metal implants can migrate or need to be removed. However, this technique is not appropriate for comminuted lateral acromion fractures or fractures which traverse into the medial aspect of the acromion. The medial aspect of the acromion and Neviaser's portal region is the anchor point for the Nice Loops.

**TABLE 2:** Advantages and Disadvantages

Advantages	Disadvantages
Multiple suture tapes allow for distribution of force while avoiding excessive bulk of plates or buttons	The current technique relies on the intact medial bone of the acromion. If a patient has osteoporosis or a fracture line traverses into the medial aspect of the acromion, this technique may put undue stress and could propagate fracture or lose reduction postoperatively
Open reduction and internal fixation without the use of plates or screws obviates the need for hardware removal in the future	In comminuted acromial fracture fragments, this technique may lead to loss of fixation and failure

## DISCLOSURES

The authors (B.M.P., J.W.G.) declare the following financial interests/personal relationships which may be considered as potential competing interests: B.M.P. reports a relationship with Stryker Orthopaedics that includes consulting or advisory. J.W.G. reports financial support provided by Stryker Orthopaedics. The other authors (G.V.C., C.J.C., M.C.M., R.T.C., M.F.B., C.L., J.V.N.) declare that they have no known competing financial interests or personal relationships that could have appeared to influence the work reported in this article.

## References

[1] Hardegger FH , Simpson LA , Weber BG. The operative treatment of scapular fractures. J Bone Joint Surg Br. 1984;66:725‐731. 6501369 10.1302/0301-620X.66B5.6501369

[2] Cole PA . Scapula fractures. Orthop Clin North Am. 2002;33:1‐18, vii. 11832310 10.1016/s0030-5898(03)00069-5

[3] van Noort A . Scapular fractures. In: Bucholz RW , Heckman JD , Court‐Brown CM , eds. Koval KJ, Tornetta III P, Wirth MA, associate editors. Rockwood and Green's fractures in adults. Philadelphia, PA: Lippincott Williams & Wilkins, 2006;1‐19.

[4] McGahan JP , Rab GT , Dublin A . Fractures of the scapula. J Trauma. 1980;20:880‐883. 6252325 10.1097/00005373-198010000-00011

[5] Nyffeler RW , Altioklar B , Bissig P . Causes of acromion and scapular spine fractures following reverse shoulder arthroplasty: A retrospective analysis and literature review. Int Orthop. 2020;44:2673‐2681. 32995915 10.1007/s00264-020-04813-5 PMC7679357

[6] Lapner PC , Uhthoff HK , Papp S . Scapula fractures. Orthop Clin North Am. 2008;39:459‐474, vi. 18803976 10.1016/j.ocl.2008.06.004

[7] Ogawa K , Matsumura N , Yoshida A , Inokuchi W . Nonunion of the so‐called acromion: A systematic review with consideration of the terminology. Arch Orthop Trauma Surg. 2023;143:5727‐5740. 37314525 10.1007/s00402-023-04912-z PMC10449677

[8] Hess F , Zettl R , Welter J , Smolen D , Knoth C . The traumatic acromion fracture: Review of the literature, clinical examples and proposal of a treatment algorithm. Arch Orthop Trauma Surg. 2019;139:651‐658. 30671623 10.1007/s00402-019-03126-6

[9] Kuhn JE , Blasier RB , Carpenter JE . Fractures of the acromion process: A proposed classification system. J Orthop Trauma. 1994;8:6‐13. 8169698 10.1097/00005131-199402000-00002

[10] Moverman MA , Menendez ME , Mahendraraj KA , Polisetty T , Jawa A , Levy JC . Patient risk factors for acromial stress fractures after reverse shoulder arthroplasty: A multicenter study. J Shoulder Elbow Surg. 2021;30:1619‐1625. 33038496 10.1016/j.jse.2020.09.012

[11] Anavian J , Wijdicks CA , Schroder LK , Vang S , Cole PA . Surgery for scapula process fractures: Good outcome in 26 patients. Acta Orthop. 2009;80:344‐350. 19857183 10.3109/17453670903025394 PMC2823212

[12] Boileau P , Alami G , Rumian A , Schwartz DG , Trojani C , Seidl AJ . The doubled‐suture nice knot. Orthopedics. 2017;40:e382‐e386. 27942736 10.3928/01477447-20161202-05

